# Supplementary material for: RNA-Seq Analysis Reveals CCR5 as a Key Target for CRISPR Gene Editing to Regulate In Vivo NK Cell Trafficking
Source: Cancers (Basel). 2021 Feb 19;13(4):872. doi: 10.3390/cancers13040872 (PMC7922167; doi:10.3390/cancers13040872)
Supplement: Supplementary file 1 [file cancers-13-00872-s001.pdf]

Supplementary Materials

RNA-Seq Analysis Reveals CCR5 as a Key Target for CRISPR Gene Editing to Regulate NK Cell Trafficking In Vivo

Emily R. Levy, Joseph A. Clara, Robert N. Reger, David S.J. Allan and Richard W. Childs

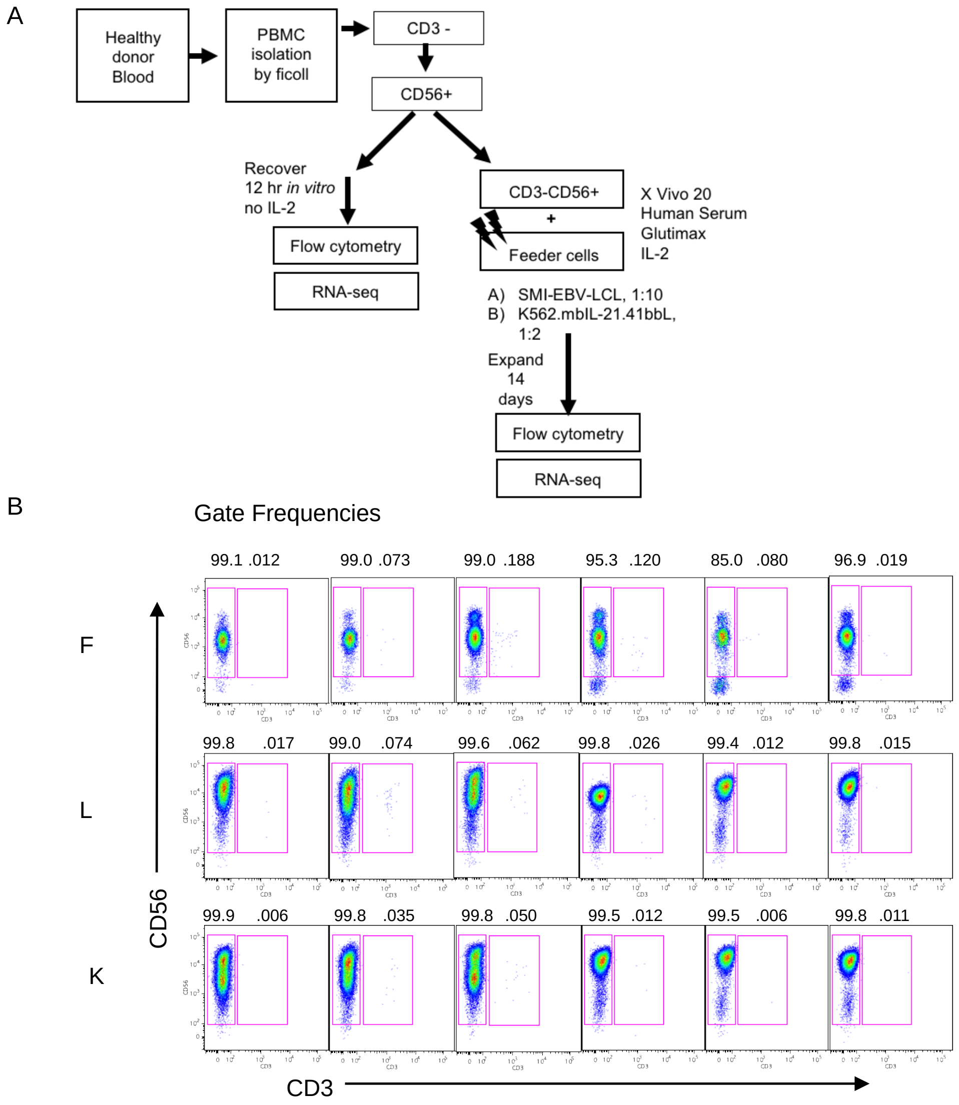

Figure S1. (**A**) Experiment outline of analyzing transcriptional and phenotypic changes in NK cells due to ex vivo expansion with feeder cells. (**B**) The frequency of viable CD56⁺CD3⁻ cells and CD3 contamination within the samples that were utilized for phenotype and RNA-seq experiments. Fresh (F) NK cells were isolated from healthy donor buffy coats (n = 6) and then expanded with either EBV-LCL (L) or GE-K562 (K) feeder cells.

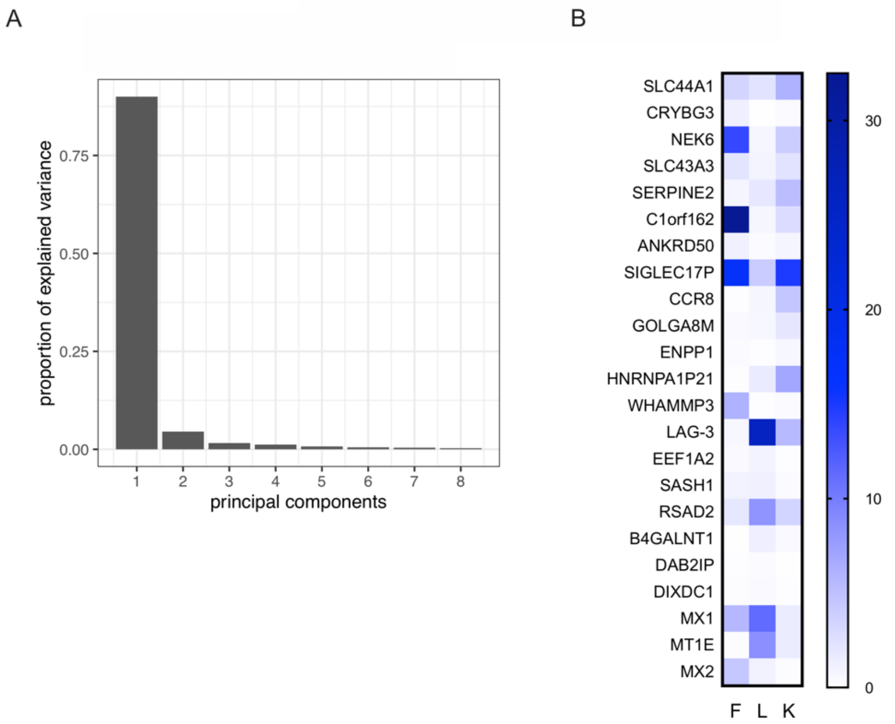

Figure S2. (**A**) Scree plot for PCA of the RNA-sequenced NK cell populations. (**B**) Heat map representing differential expression of significant DEGs between LCL and GE-K562 expanded NK cells (FPKM).

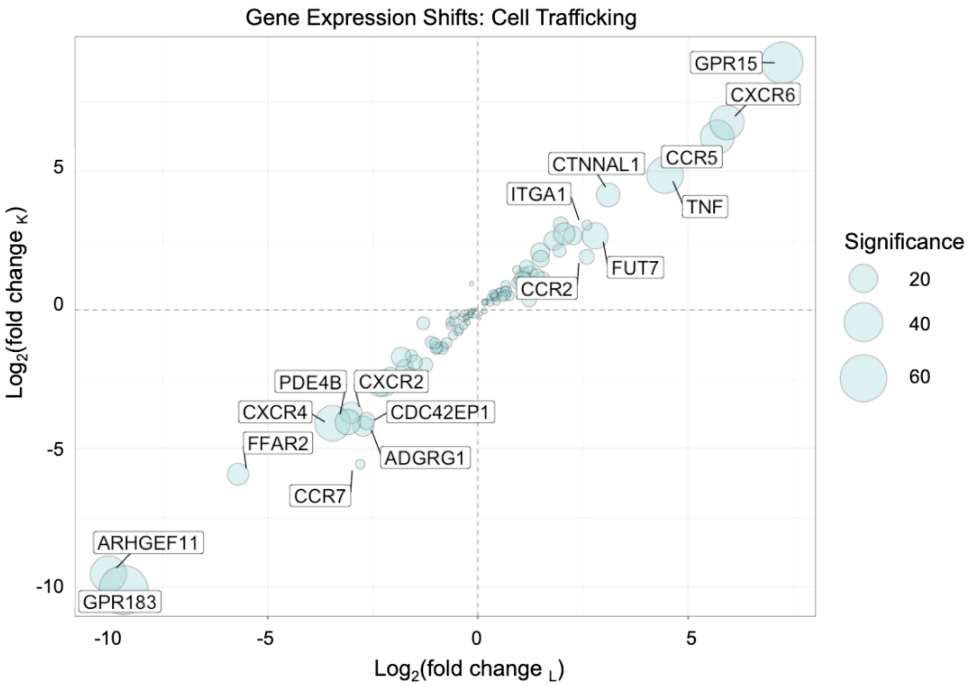

Figure S3. Bubble plots that displays genes that are included in supplemental Table 1; graphical organization is the same as in Figure 2A. The genes that are labeled have a $\log_2$(fold change) value of >3 or <−3. Significance is reported as $-\log_{10}$(adjusted *p* value).

© 2021 by the authors. Submitted for possible open access publication under the terms and conditions of the Creative Commons Attribution (CC BY) license (http://creativecommons.org/licenses/by/4.0/).
